# Analyzing the association between fish consumption and osteoporosis in a sample of Chinese men

**DOI:** 10.1186/s41043-017-0088-y

**Published:** 2017-04-19

**Authors:** Xia Li, Tao Lei, Zihui Tang, Jingcheng Dong

**Affiliations:** 1 Department of Integrative Medicine, Huashan Hospital, Fudan University, No.12 Urumqi Middle Road, 200040 Shanghai, China; 2 The Institutes of Integrative Medicine, Fudan University, Shanghai, China; 3 Department of Endocrinology and Metabolism, Shanghai Tongji Hospital, Tongji University School of Medicine, Shanghai, China

**Keywords:** Frequency, Fish food consumption, Osteoporosis, Chinese men, Association

## Abstract

**Background:**

The main purpose of this study was to estimate the associations between frequency of fish food consumption and osteoporosis (OP) in general Chinese men.

**Methods:**

We conducted a large-scale, community-based, cross-sectional study to investigate the associations by using self-report questionnaire to access frequency of fish food intake. A total of 1092 men were available for data analysis in this study. Multiple regression models controlling for confounding factors to include frequency of fish food consumption variable were performed to investigate the relationships for OP.

**Results:**

Positive correlations between frequency of fish food consumption and *T* score were reported (*β* = 0.084, *P* value = 0.025). Multiple regression analysis indicated that the frequency of fish food consumption was significantly associated with OP (*P* < 0.05 for model 1 and model 2). The men with high frequency of fish food consumption had a lower prevalence of OP.

**Conclusions:**

The findings indicated that frequency of fish food consumption was independently and significantly associated with OP. The prevalence of OP was less frequent in Chinese men preferring fish food habits.

**Trial registration:**

ClinicalTrials.gov Identifier: NCT02451397 retrospectively registered 28 May 2015.

## Background

Osteoporosis (OP) is a metabolic syndrome characterized by reduced bone mass and microarchitectual deterioration of bone tissue, accompanied by a high risk of fracture, particularly in areas such as the hip, vertebral, forearm, pelvis, calcaneus, ribs, and so on [1]. With the emergence of an aging population, the prevalence of OP is rapidly growing and poses a serious threat to human health, especially in Asia. For example, it is predicted that the number of global hip fractures will reach 6.26 million in 2050, about 30% of which will occur in Asia, most notably in China. It is expected that the burden of hip fracture may be shifting from the West to the East, specifically in urban areas [2].

In addition to genetic predisposition, nutrition and lifestyle play key roles in the maintenance of bone health. Smoking, alcoholism, being bedridden, calcium and vitamin D insufficiency, and a high-salt diet may have negative effects on bone health, whereas physical activity and sun exposure may help to prevent OP. Recent studies have indicated that a diet rich in n-3 polyunsaturated fatty acids (n-3PUFAs) is associated with higher bone mineral density (BMD) and decreased bone loss [3]. Fish, which constitutes a major food group in diets throughout the world, contains high levels of n-3PUFAs, high-quality protein, and vitamins and essential minerals, and thus may be beneficial to bone health. However, these conclusions are still controversial. Some studies have reported that fish consumption can in fact reduce the risk of bone fracture [4]. On the contrary, other studies have indicated that fish consumption has no association with BMD or hip fracture risk [5]. Moreover, most studies were conducted in countries other than China, and very little research has focused on the bone health of the Chinese population.

Our previous association analyses for OP in Chinese postmenopausal women showed the relationships among meat consumption, coffee consumption, coronary artery disease and this outcome [6–8]. Additionally, we analyzed the associations among education level, rheumatoid arthritics and OP in a sample of Chinese men [9, 10]. Recently, it was reported that one in four men will suffer from osteoporosis-related fracture in their lifetime, and that hip fracture contributes greatly to morbidity and mortality in men [11]. Therefore, it is critical to investigate the risk factors and preventative measures associated with OP in men. The purpose of this study was to investigate the association between the frequency of fish consumption and OP in a sample of Chinese men using a self-reported questionnaire.

## Methods

### Study population

As we mentioned earlier [8–10], a risk-factor study for OP was conducted in a random sample of the Chinese population. Participants aged 30–90 years were recruited from rural and urban communities in Shanghai. More than 3000 participants (both male and female) were invited to a screening visit between 2011 and 2013. Written consent was obtained from all patients before the study, which was performed in accordance with the ethical standards in the Declaration of Helsinki, and approved by the Medicine Ethical Committee of the Huashan Hospital. Some participants with chronic diseases and conditions that might potentially affect bone mass, structure, or metabolism were excluded. Briefly, the exclusion criteria were detailed in our previous studies. A total of 1092 Chinese men were available to data analysis.

### Data collection

As we mentioned earlier [9, 10], all study subjects underwent complete clinical baseline characteristics evaluation, which included a physical examination and response to a structured, nurse-assisted, self-administrated questionnaire to collect information on age, gender, residential region, visit date, family history, lifestyle, dietary habits, physical activity level during leisure time, use of vitamins and medications, smoking, alcohol consumption, and self-reported medical history. Smoking, alcohol consumption, regular exercise, education and dietary habit were categorized as mentioned in previous studies. In addition, the definitions of HTN, body mass index (BMI), and diabetes mellitus (DM) were detailed earlier, respectively.

To determine frequency of fish food preference, the participants were asked, “How often you eat fish food?” The possible answers were “seldom,” “once or twice per week,” “once per 2 day,” or “every day,” and the answers were taken as a subjective assessment. To answer the question, the participants were required to decide two issues based on their impressions: (1) whether or not the consumed foods were actually fish; and (2) the frequency with which they consumed fish foods.

### The study outcomes

As we mentioned earlier [9, 10], the bone mineral density (BMD g/cm^2^) was measured at calcaneus by standardized quantitative ultrasound (QUS, Hologic Inc., Bedford, MA, USA) utilizing *T* scores based on WHO criteria [12], which were obtained from the automated equipment. The diagnosis of OP was detailed earlier.

### Statistical analysis

Continuous variables were analyzed to determine whether they followed normal distributions, using the Kolmogorov-Smirnov Test. Variables that were not normally distributed were log-transformed to approximate a normal distribution for analysis. Results are described as mean ± SD or median, unless stated otherwise. Differences in variables among subjects grouped by frequency of fish food intake were determined by one-way-analysis of variance. Among groups, differences in properties were detected by *χ* ^2^ analysis.

Univariate regression analysis was performed to determine variables associated with outcomes (*T* score or OP). Additionally, multivariable regression (MR) was performed to control potential confounding factors and determine the independent contribution of variables to outcomes (*T* score or OP). For the associations analysis, there model have been developed. In model 1, frequency of fish food intake were categorized by group 1: seldom, group 2: once or twice per week, group 3: once per 2 days, and group 4: always. In model 2: frequency of fish food intake were categorized by group 1: seldom, group 2: sometimes, group 3: always. In model 3: frequency of fish food intake were categorized by low frequency and high frequency groups. Results were analyzed using the Statistical Package for Social Sciences for Windows, version 16.0 (SPSS, Chicago, IL, USA). Tests were two-sided, and a *P* value of <0.05 was considered significant. Odds ratios (OR) with 95% confidence intervals (CI) were calculated for the relative risk of frequency of fish food intake with the outcome of OP.

## Results

### Clinical characteristics of subjects

The clinical baseline characteristics of the 1092 Chinese male subjects were detailed earlier [9, 10] and listed in Table 1. In the total sample, the mean age was 64.11 years, and the mean height and weight were 168.16 cm and 67.96 kg, respectively. The proportions of subjects having current smoking and alcohol habits were 36.39 and 30.58%, respectively. The prevalence of HTN, coronary artery disease (CAD), DM, Gout, and Rheumatoid arthritis (RA) were 45.78, 10.29, 9.73, 3.56, and 3.43%, respectively. An average *T* score of −1.23 was reported and the prevalence of OP was 8.79% in our study sample. There were significant differences in age, smoking habits, exercise and education among groups according to frequency of fish food intake (*P* value <0.05 for all). Significant differences in *T* score and the prevalence of OP among the four groups (*P* value =0.024 for *T* score and 0.028 for the prevalence of OP).

**Table 1:** Baseline characteristics of subjects

Variable	Total sample	Frequency of fish food intake ^a^				*P* value
		Group 1	Group 2	Group 3	Group 4	
Demographic information						
*N*	1092	93	671	241	87	–
Age	64.11 ± 9.77	64.1 ± 10.32	64.62 ± 9.81	63.71 ± 9.53	61.28 ± 9.21	0.023
Height	168.16 ± 5.61	167.97 ± 6.07	167.97 ± 4.75	167.35 ± 6.73	170.58 ± 6.48	0.440
Weight	67.96 ± 11.94	65.78 ± 9.91	67.23 ± 9.21	69.4 ± 19.55	71.42 ± 7.71	0.581
Lifestyle						
Smoking	397 (36.39%)	46 (49.46%)	237 (35.37%)	80 (33.2%)	34 (39.08%)	0.037
Drink intake	333 (30.58%)	30 (32.26%)	192 (28.66%)	78 (32.5%)	33 (38.37%)	0.244
Excise	705 (64.56%)	50 (53.76%)	423 (63.04%)	175 (72.61%)	57 (65.52%)	0.006
Education	299 (27.38%)	25 (26.88%)	170 (25.34%)	75 (31.12%)	29 (33.33%)	<0.001
Oil	20.22 ± 10.13	21.25 ± 10.74	20.13 ± 9.76	19.55 ± 10.37	21.64 ± 11.48	0.286
Medical history						
HTN	494 (45.78%)	38 (40.86%)	311 (46.77%)	112 (47.66%)	33 (38.37%)	0.333
CAD	108 (10.29%)	9 (10.11%)	66 (10.09%)	24 (10.62%)	9 (11.11%)	0.990
DM	104 (9.73%)	8 (8.7%)	68 (10.3%)	20 (8.58%)	8 (9.52%)	0.870
Gout	38 (3.56%)	3 (3.3%)	19 (2.9%)	12 (5.11%)	4 (4.65%)	0.424
RA	37 (3.43%)	5 (5.38%)	21 (3.17%)	8 (3.38%)	3 (3.49%)	0.753
Medicine history						
Vitamin C yes %	115 (10.53%)	11 (11.83%)	65 (9.69%)	24 (9.96%)	15 (17.24%)	0.178
Vitamin D yes %	29 (2.66%)	1 (1.08%)	20 (2.98%)	4 (1.66%)	4 (4.6%)	0.338
Outcome						
*T* score	−1.23 ± 0.91	−1.36 ± 1.05	−1.23 ± 0.92	−1.26 ± 0.82	−0.97 ± 0.89	0.024
OP	96 (8.79%)	15 (15.96%)	61 (9.10%)	15 (6.22%)	5 (5.75%)	0.028

*HTN* hypertension, *CAD* coronary artery disease, *DM* diabetes mellitus, *RA* rheumatoid arthritis, *OP* osteoporosis

^a^Frequency of fish food intake were categorized by group 1: seldom, group 2: once or twice per week, group 3: once per 2 days, and group 4: always

### Univariate analysis for *T* score and OP

As we partly mentioned earlier [9, 10], univariate linear regression analyses were developed to include demographical information, medical history, and lifestyle to estimate the association of various clinical factors and *T* score. The variables age, exercise, education, and fish food intake were significantly associated with the *T* score.

**Fig. 1:**
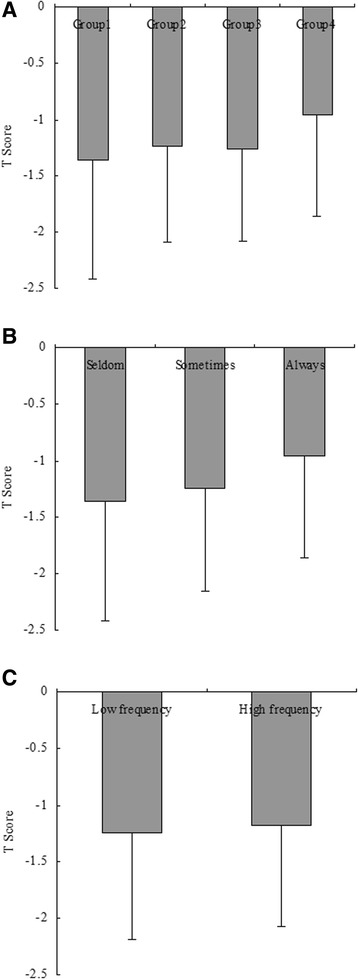
Comparison of *T* score among groups according to frequency of fish food intake. **a** The results of comparison of *T* score among groups according to Model 1 (Model 1: frequency of fish food intake were categorized by group 1: seldom, group 2: once or twice per week, group 3: once per 2 days, and group 4: always). The mean *T* score was −1.36, −1.23, −1.26, and −0.97 in the four groups, respectively. There were significantly differences among the three groups (*P* = 0.024). **b** The results of comparison of *T* score among groups according to Model 2 (Model 2: frequency of fish food intake were categorized by group 1: seldom, group 2: sometimes, group 3: always). The mean *T* score was −1.36, −1.24, and −0.97 in the three groups, respectively. There were significantly differences among the three groups (*P* = 0.010). **c** The results of comparison of *T* score between groups according to Model 3 (Model 3: frequency of fish food intake were categorized by low frequency and high frequency groups). The mean *T* score was −1.25 and −1.18 in the two groups, respectively. There were no significant differences between the two groups (*P* = 0.231)

The comparison of *T* scores among groups according to frequency of fish food intake (categorized by group 1: seldom, group 2: once or twice per week, group 3: once per 2 days, and group 4: always) revealed that the mean *T* score was −1.36, -1.23, −1.26, and −0.97 in the four groups, respectively (Fig. 1a). There were significant differences among the four groups (*P* = 0.024). Additionally, there were significant differences among groups according to model 2 (Fig. 1b, *P* = 0.010), while no significant differences among groups according to model 3 was reported (Fig. 1c, *P* = 0.231). Univariate analysis demonstrated a positive correlation between frequency of fish food intake and *T* score.

**Fig. 2:**
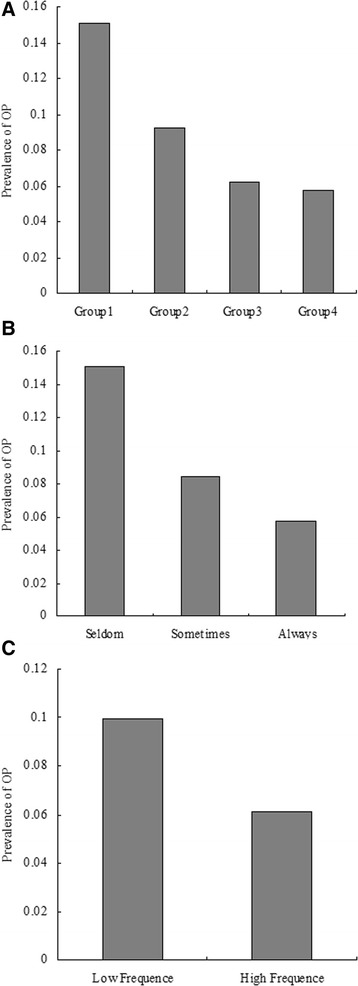
Comparison of prevalence of osteoporosis among groups according to frequency of fish food intake. **a** The results of comparison of prevalence of osteoporosis among groups according to Model 1 (Model 1: frequency of fish food intake were categorized by group 1: seldom, group 2: once or twice per week, group 3: once per 2 days, and group 4: always). The prevalence of osteoporosis was 15.96, 9.10, 6.22, and 5.75% in the four groups, respectively. There were significantly differences among the four groups (*P* = 0.028 and *P* value for trend =0.014). **b** The results of comparison of prevalence of osteoporosis among groups according to Model 2 (Model 2: frequency of fish food intake were categorized by group 1: seldom, group 2: sometimes, group 3: always). The prevalence of osteoporosis was 15.96, 8.34, and 5.75% in the three groups, respectively. There were significantly differences among the three groups (*P* = 0.027 and *P* value for trend =0.036). **c** The results of comparison of prevalence of osteoporosis between groups according to Model 3 (Model 3: frequency of fish food intake were categorized by low frequency and high frequency groups). The prevalence of osteoporosis was 9.95 and 6.10% between the two groups, respectively. There were significantly differences between the two groups (*P* = 0.039 and *P* value for trend =0.040)

As we partly mentioned earlier [9, 10], univariate logistic analyses were performed to evaluate associations with OP. The results indicate that age, RA, alcohol intake, exercise, education, and frequency of fish food intake were significantly associated with OP (*P* < 0.05 for all). The comparison of prevalence of OP among groups according to model 1 reported that the prevalence of OP was 15.96, 9.10, 6.22, and 5.75% in the four groups, respectively (Fig. 2a). There were significant differences among the four groups (*P* = 0.028). In addition, significant differences among groups according to model 2 and model 3 were reported (Fig. 2b and c, *P* = 0.027 for model 2 and *P* = 0.039 for model 3). Univariate analysis demonstrates a negative correlation between frequency of fish food intake and OP.

### Multiple variable analysis for *T* score and OP

Multivariate linear regression analyses were developed to include frequency of fish food intake and the outcome of *T* score. After adjustment for relevant potential confounding factors, the multivariate linear regression analyses detected significant associations (*β* = 0.074, *P* = 0.048, 95% CI 0.002–0.148 for model 1; and *β* = 0.161, *P* = 0.018, 95% CI 0.028–0.295 for model 2, Table 2). No significant associations were reported in model 3 (*P* = 0.402).

**Table 2:** Multiple variables linear regression analysis for the associations between frequency of fish food intake and *T* score

Model	Variable	*β*	SE	*P* value	95% CI for *β*
Model 1	Frequency of fish food intake	0.074	0.037	0.050	0.002–0.148
Model 2	Frequency of fish food intake	0.161	0.068	0.018	0.028–0.295
Model 3	Frequency of fish food intake	0.051	0.060	0.402	−0.068–0.169

Model 1: frequency of fish food intake were categorized by group 1: seldom, group 2: once or twice per week, group 3: once per 2 days, and group 4: always; Model 2: frequency of fish food intake were categorized by group 1: seldom, group 2: sometimes, group 3: always; Model 3: frequency of fish food intake were categorized by low frequency and high frequency groups; and all models adjusted for age, smoking, alcohol intake, education, exercise and medical history

Multivariate logistic regression analyses were employed to evaluate the association between frequency of fish food intake and the OP outcome. After adjustment for relevant potential confounding factors, the multivariate logistic regression analyses detected significant associations (*P* = 0.042 for model 1; and *P* = 0.046 for model 2, Table 3). In participants with frequency of fish food intake, the OR for OP was 0.709 in model 1 (95% CI 0.509–0.987). No significant associations was found in model 3 (*P* = 0.193).

**Table 3:** Multiple variables logistic regression analysis for associations between frequency of fish food intake and osteoporosis

Model	Variable	*β*	S.E.	*P* value	OR	95%CI
Model 1	Frequency of fish food intake	−0.344	0.169	0.042	0.709	0.509–0.987
Model 2	Frequency of fish food intake	−0.556	0.279	0.046	0.573	0.332–0.990
Model 3	Frequency of fish food intake	−0.351	0.270	0.193	0.704	0.415–1.194

Model 1: frequency of fish food intake were categorized by group 1: seldom, group 2: once or twice per week, group 3: once per 2 days, and group 4: always; Model 2: frequency of fish food intake were categorized by group 1: seldom, group 2: sometimes, group 3: always; Model 3: frequency of fish food intake were categorized by low frequency and high frequency groups; and all models adjusted for age, smoking, alcohol intake, education, exercise and medical history

## Discussion

In this community-based, cross-sectional study, we found that frequency of fish consumption was positively associated with calcaneus BMD in Chinese men. We used a self-report questionnaire to estimate the fish consumption due to its convenience for large-scale, cross-sectional study. Fish is the major source of animal protein consumed in traditional Chinese diets. Our findings suggest that increasing the frequency of fish consumption may be an effective and economic way to prevent OP.

Our results are consistent with those of previous studies. For instance, Julian et al. found that greater fish consumption was associated with greater bone mass of the phalanges among pre-menopausal Spanish women [13]. Farina et al. also examined the association between fish consumption and hip bone mineral density cross-sectionally and longitudinally in the Framingham Osteoporosis Study, which indicated that both men and women with high fish consumption demonstrated greater mean baseline femoral neck BMD, and that the consumption of fish was inversely associated with bone loss at the femoral neck over a period of 4 years [14]. Furthermore, the same study showed that BMD was significantly higher among participants living in a fishing village than among those living in a mountain village with less access to fish [15]. In contrast to this research, the Cardiovascular Health Study reported that there were no associations between fish consumption and fracture risk [5]. Furthermore, the effect of fish consumption on bone health may vary among fish species. A case-control study showed significantly dose-dependent inverse correlations between the risk of hip fracture and intake of saltwater fish, as opposed to freshwater fish [16]. Similar results were reported in Hong Kong [17]. In fact, most studies have proven that saltwater fish are more effective in preventing OP than freshwater fish. While our study did not distinguish between the two fish types, our results suggested that frequency of fish consumption in general was independently and significantly associated with OP among Chinese men.

Fish is the predominant dietary source of n-3PUFAs, eicosapentaenoic acid (EPA), and docosahexaenoic acid (DHA). In the past several decades, a large number of studies have demonstrated that n-3PUFAs may have a positive influence on bone metabolism. Further, n-3PUFAs have been positively associated with lumbar spine BMD in older adults [18]. Similar results were found in another study, which suggested that a higher red blood cell count, a-linolenic acid, as well as EPA and total n-3PUFAs, may contribute to lower hip fracture risk [19]. Still, other studies conducted on animals have found that incorporating fish oil into a diet can slow the loss of bone observed following menopause and old age [20]. The mechanisms by which n-3PUFAs operate can be illustrated as follows. Firstly, they inhibit the generation and activation of osteoclasts. Specifically, dietary n-3PUFAs have been shown to decrease the expression of inflammatory cytokines (e.g., interleukin-1, interleukin-6, and tumor necrosis factors), which dose-dependently increases the expression of receptor activator NFKB ligand (RANKL) [21]. Secondly, n-3PUFAs modulate calcium balance by increasing calcium absorption and decreasing urinary calcium excretion [22]. Thirdly, they promote osteogenic differentiation by enhancing the expression of key transcription factors. In vivo and in vitro studies have indicated that dietary n-3PUFAs may up-regulate the expression of IGF-1, IGF-binding proteins, and TGF-B1, thus enhancing the differentiation of pre-osteoblasts into mature osteoblasts [23]. Furthermore, n-3PUFAs may reduce the expression of peroxisome proliferator-activated receptorγ (PPARr), a transcription factor involved in osteoblast/adipocyte commitment, thus favoring osteoblast differentiation [24].

In addition to n-3PUFAs, fish are rich in vitamin D and protein. Vitamin D deficiency is recognized as a worldwide problem for both children and adults. It plays a significant role in regulating calcium and phosphorus metabolism and inhibiting bone resorption. A study conducted in the Netherlands demonstrated that fish is the greatest modifiable contributor to the serum 25-hydroxy vitamin D concentration in a multiethnic population [25]. Fish consumption accounts for 87% of total dietary vitamin D intake in Spain, and 90.1% in Japan [26]. However, our investigation failed to demonstrate a significant correlation between fish consumption and vitamin D levels among its sample of Chinese men. The impact of dietary protein on bone remains controversial. Dietary protein is a recognized determinant of urinary calcium excretion, but it can also increase intestinal calcium absorption and improves muscle strength and mass [27]. Taken together, determining dietary protein’s relationship with fracture risk requires further investigation.

Our study has several limitations. Firstly, statistics obtained from self-reported questionnaires may not reflect the actual behavior of the participants. Additionally, we cannot establish a definite causal relationship between fish consumption and OP because it is unclear how much time has passed between exposure and outcome in this study. Additionally, as this study was based on a cross-sectional study for association analysis, it also requires a larger sample size and more geographic representation. Finally, the study’s sample was composed entirely of Chinese men, thus limiting the generalizability of our results.

## Conclusions

Our findings suggest that frequency of fish consumption was independently and significantly associated with OP in our sample. The prevalence of OP was lower in Chinese men who preferred eating fish. This study suggests that a change in dietary preference in favor of fish might be beneficial in the prevention of OP among Chinese men.

## Abbreviations

BMD: Bone mineral density
BMI: Body mass index
BM-MNC: Bone marrow-derived mononuclear cell
CAD: Coronary artery disease
CI: Confidence intervals
DEXA: Dual-energy X-ray
DHA: Docosahexaenoic acid
DM: Diabetes
EPA: Eicosapentaenoic acid
GFR: Glomerular filtration rate
HTN: Hypertension
IGF-1: Insulin-like growth factor-1
n-3PUFA: N-3polyunsaturated fatty acids
OGTT: Oral glucose tolerance test
OP: Osteoporosis
OR: Odds ratios
PPARr: Peroxisome proliferator-activated receptor γ
QUS: Quantitative ultrasound
RA: Rheumatoid arthritis
RANKL: Receptor activator of NFKB ligand
TGF-β1: Transforming growth factor-β1
